# Supplementary figures and images for: Segregation of pathways leading to pexophagy
Source: Life Sci Alliance. 2023 Feb 21;6(5):e202201825. doi: 10.26508/lsa.202201825 (PMC9944197; doi:10.26508/lsa.202201825)

Source Data: Figure 1 E: blot 1

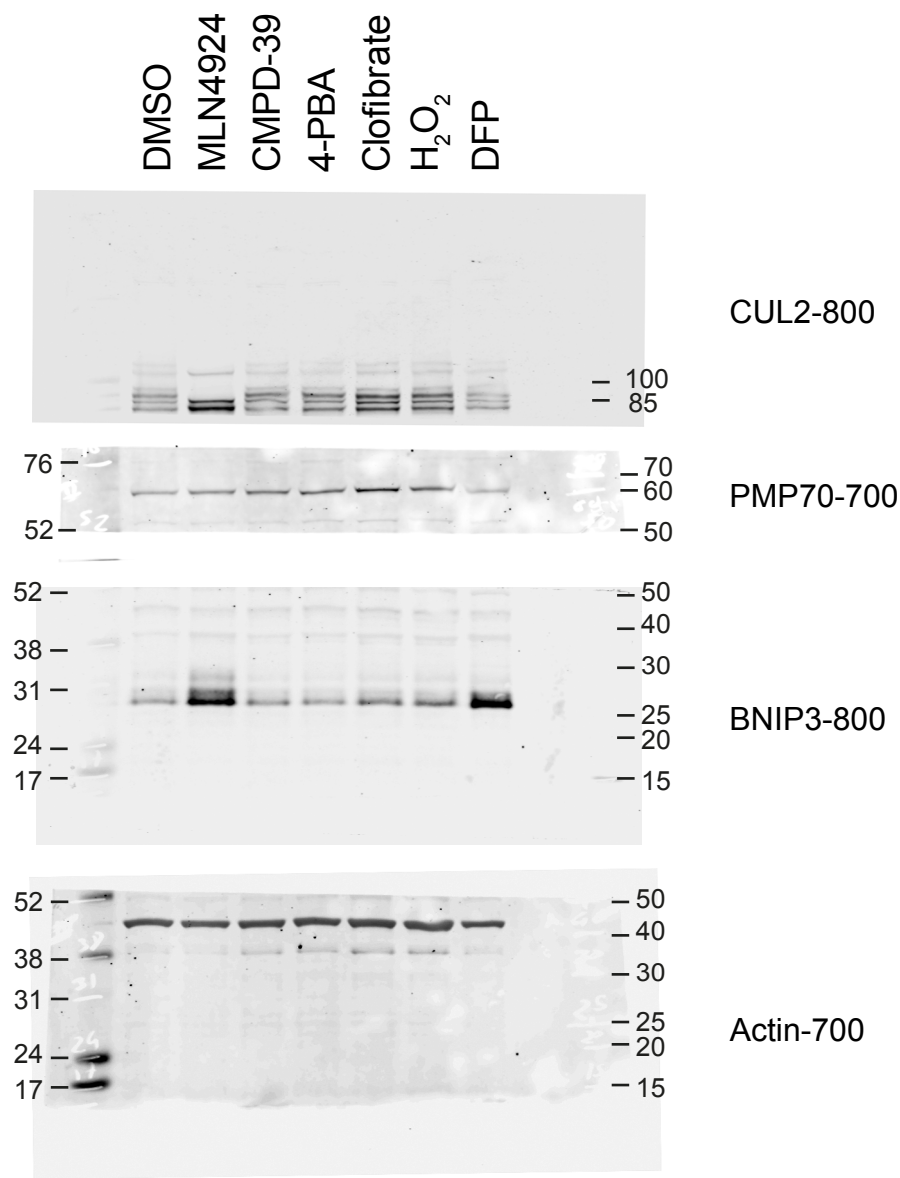

Source Data: Figure 1 E: blot 2

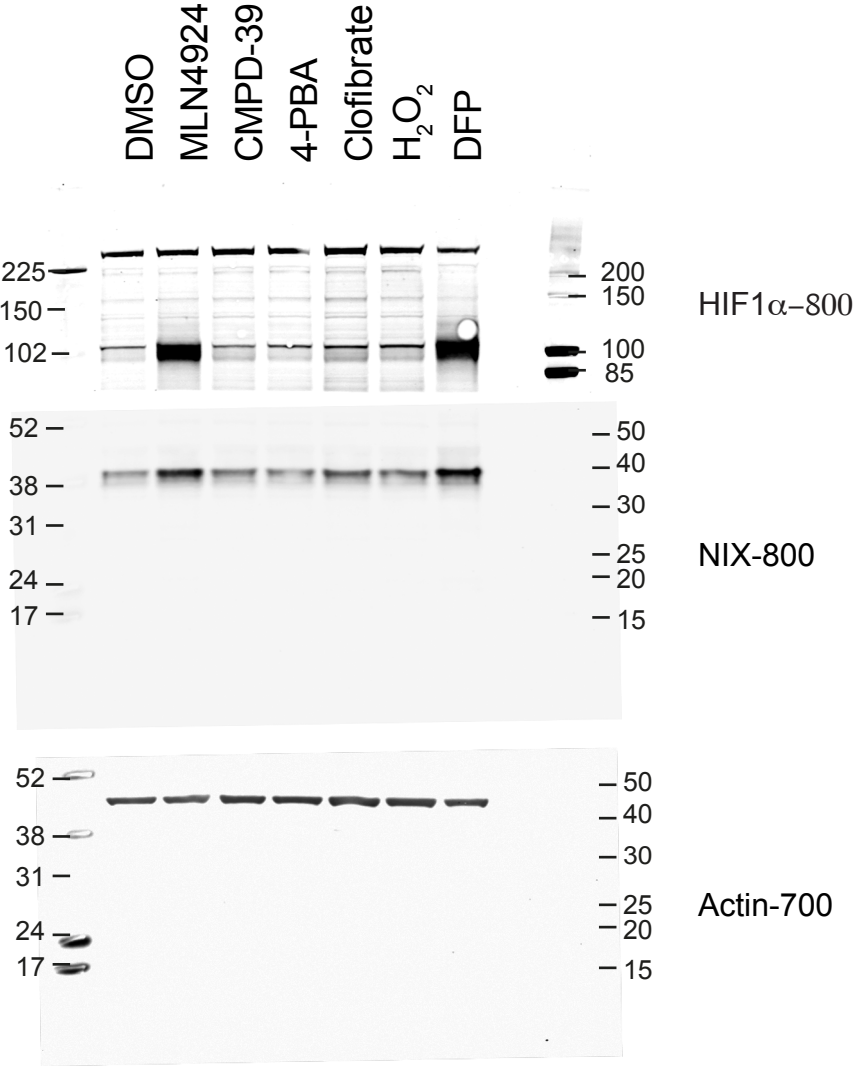

Source Data: Figure 1 E: blot 3

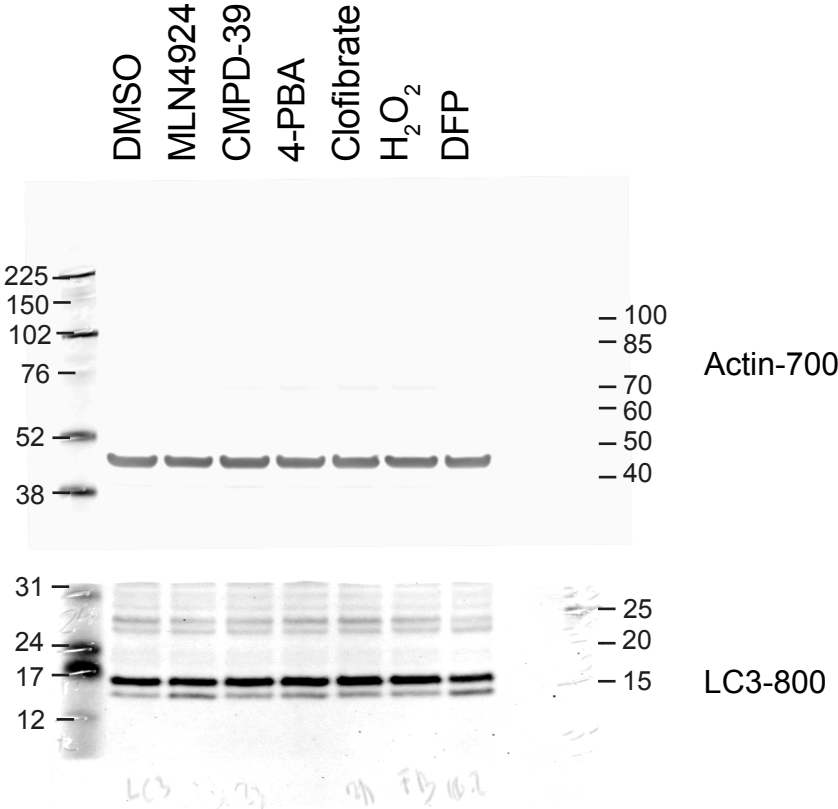

Supplement: Supplementary file 1 [file LSA-2022-01825_SdataF1.pdf]

Source Data: Figure 3 D

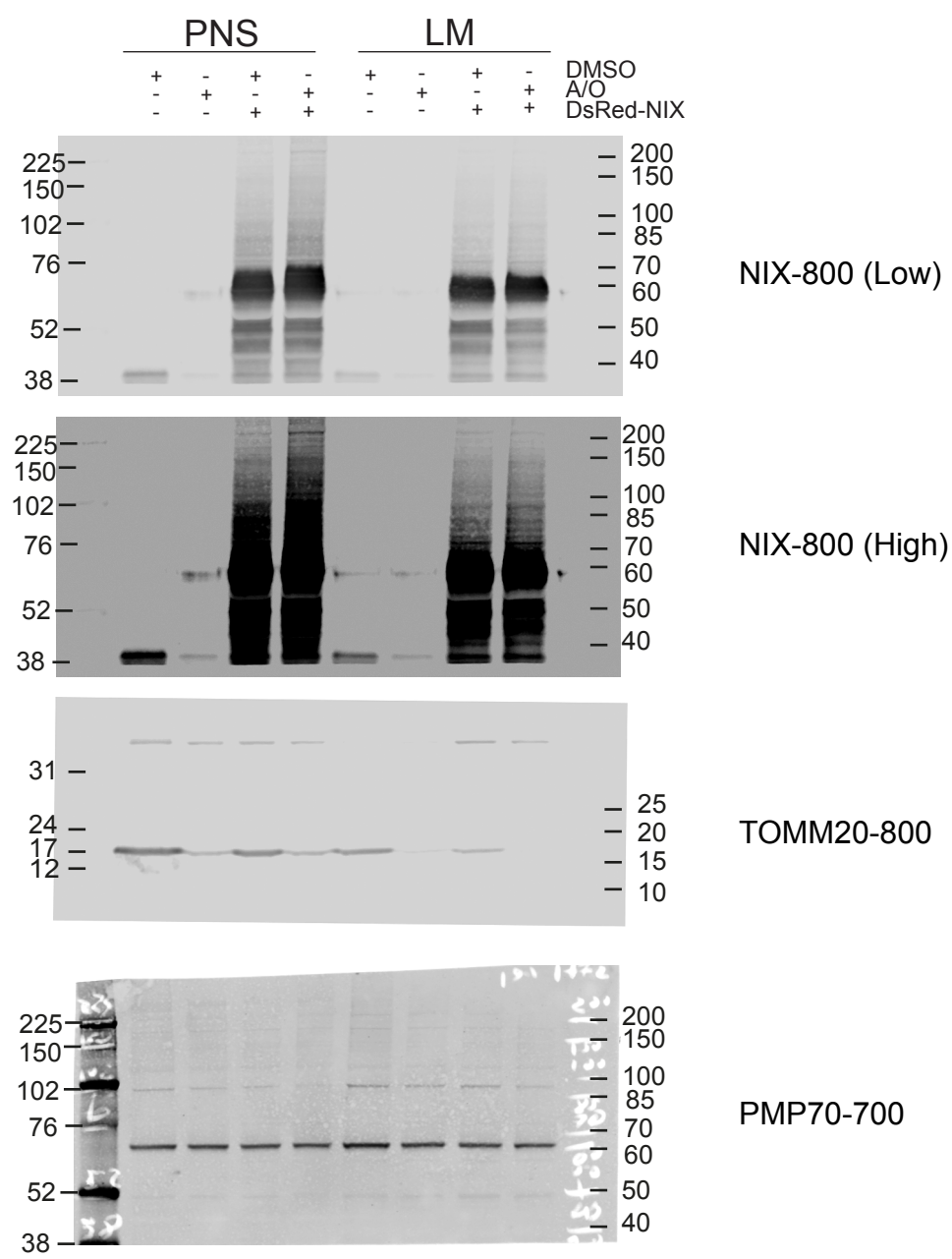

Supplement: Supplementary file 3 [file LSA-2022-01825_SdataF3.pdf]

Source Data: Figure 4 D Blot 1

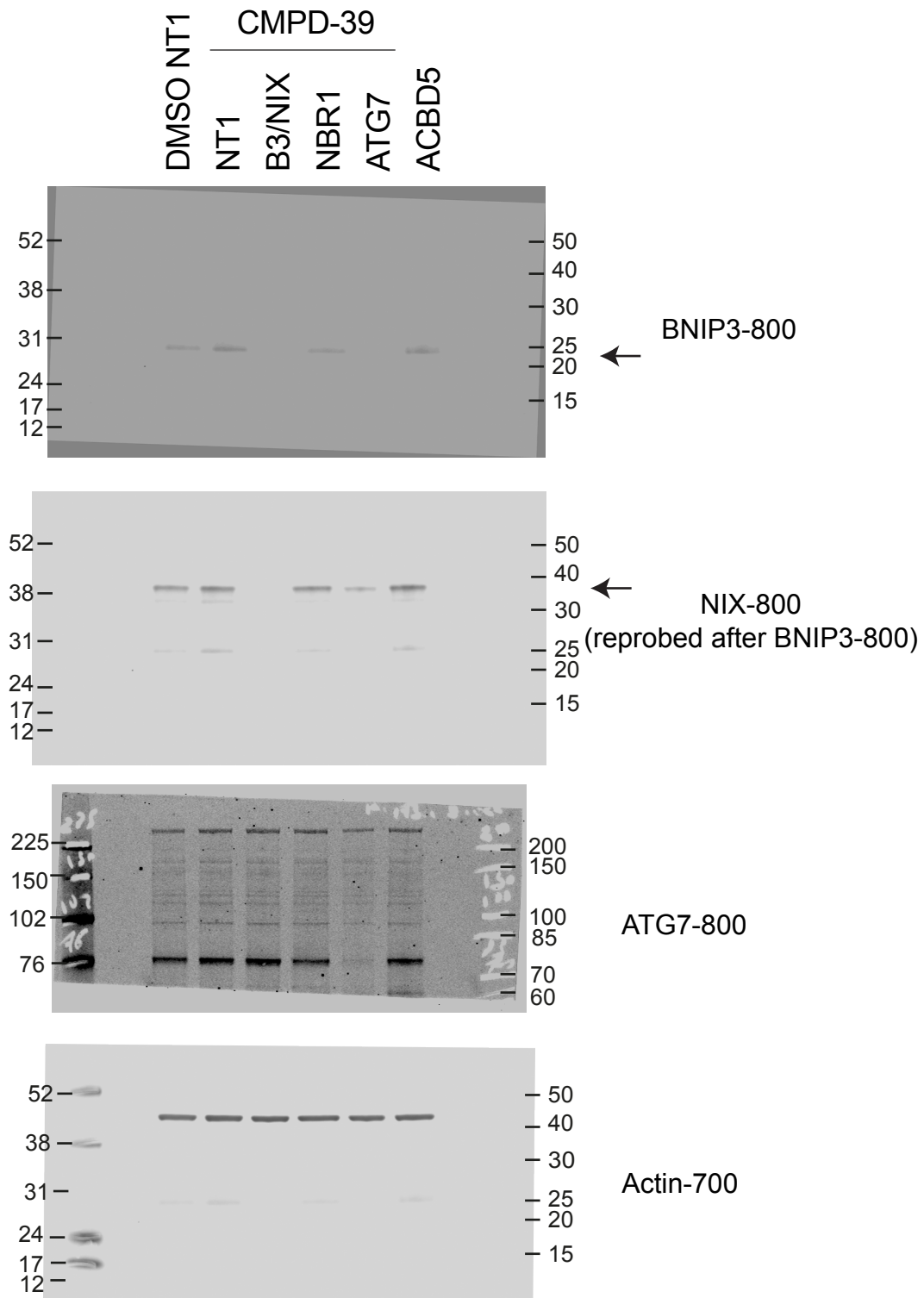

Source Data: Figure 4 D Blot 2

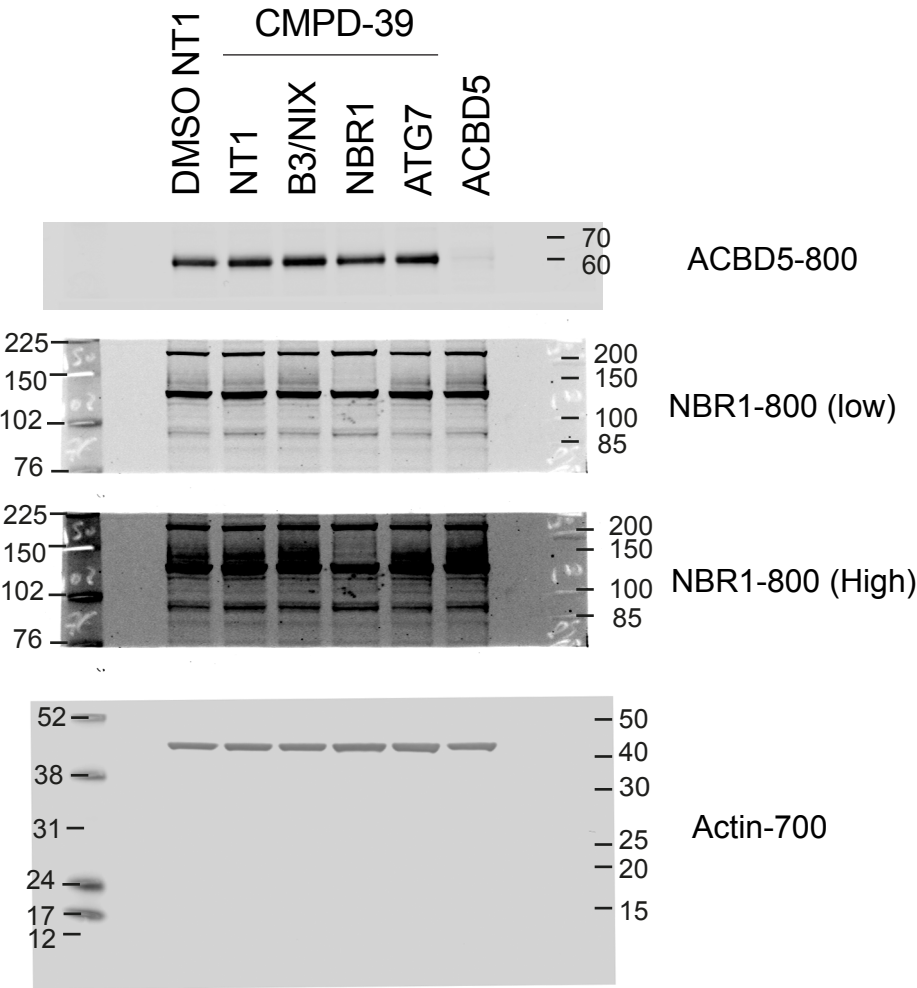

Supplement: Supplementary file 4 [file LSA-2022-01825_SdataF4.pdf]
