# Supplementary material for: Segregation of pathways leading to pexophagy
Source: Life Sci Alliance. 2023 Feb 21;6(5):e202201825. doi: 10.26508/lsa.202201825 (PMC9944197; doi:10.26508/lsa.202201825)

Source Data: Figure 2 D: Blot 1

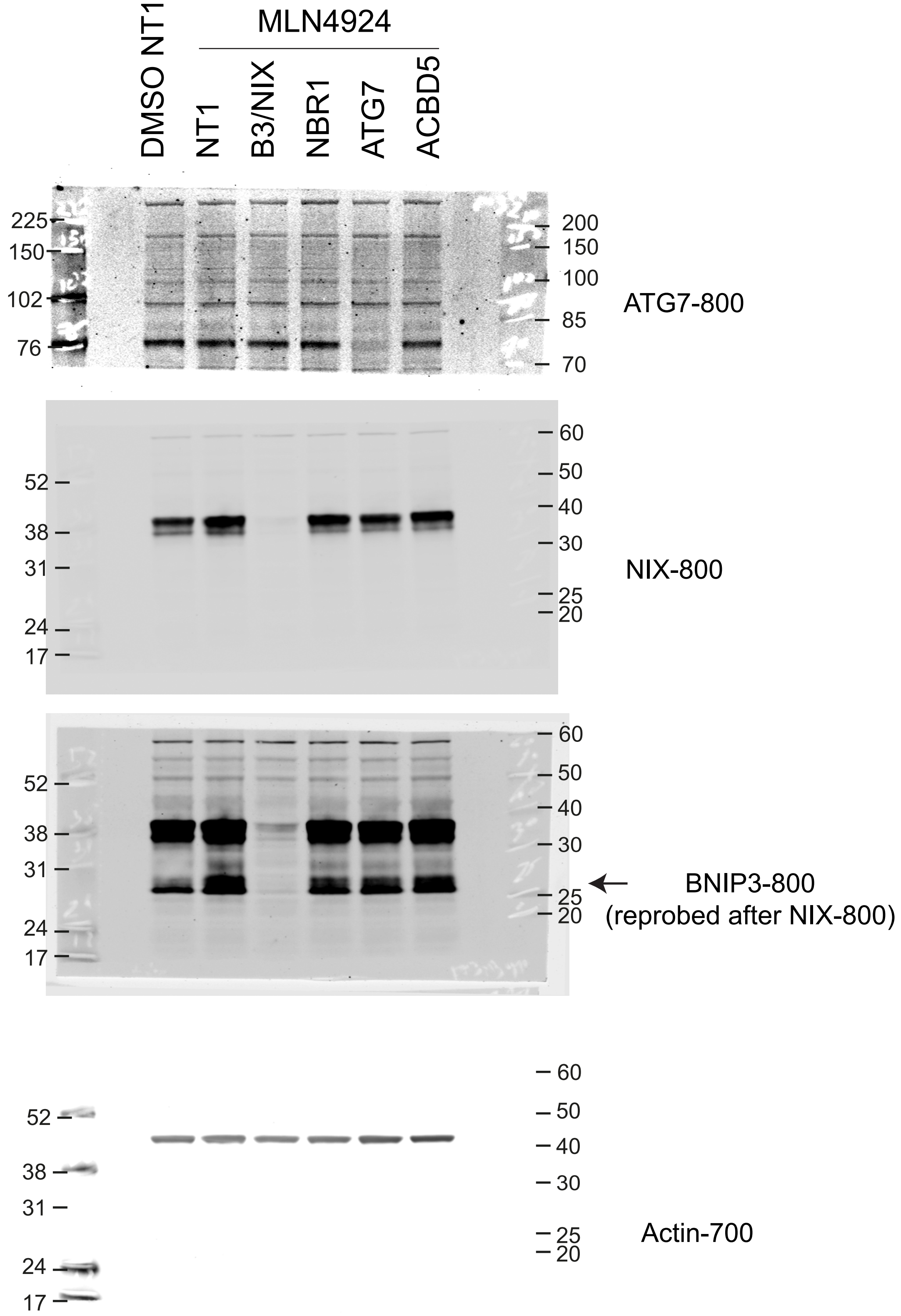

Source Data: Figure 2 D: Blot 2

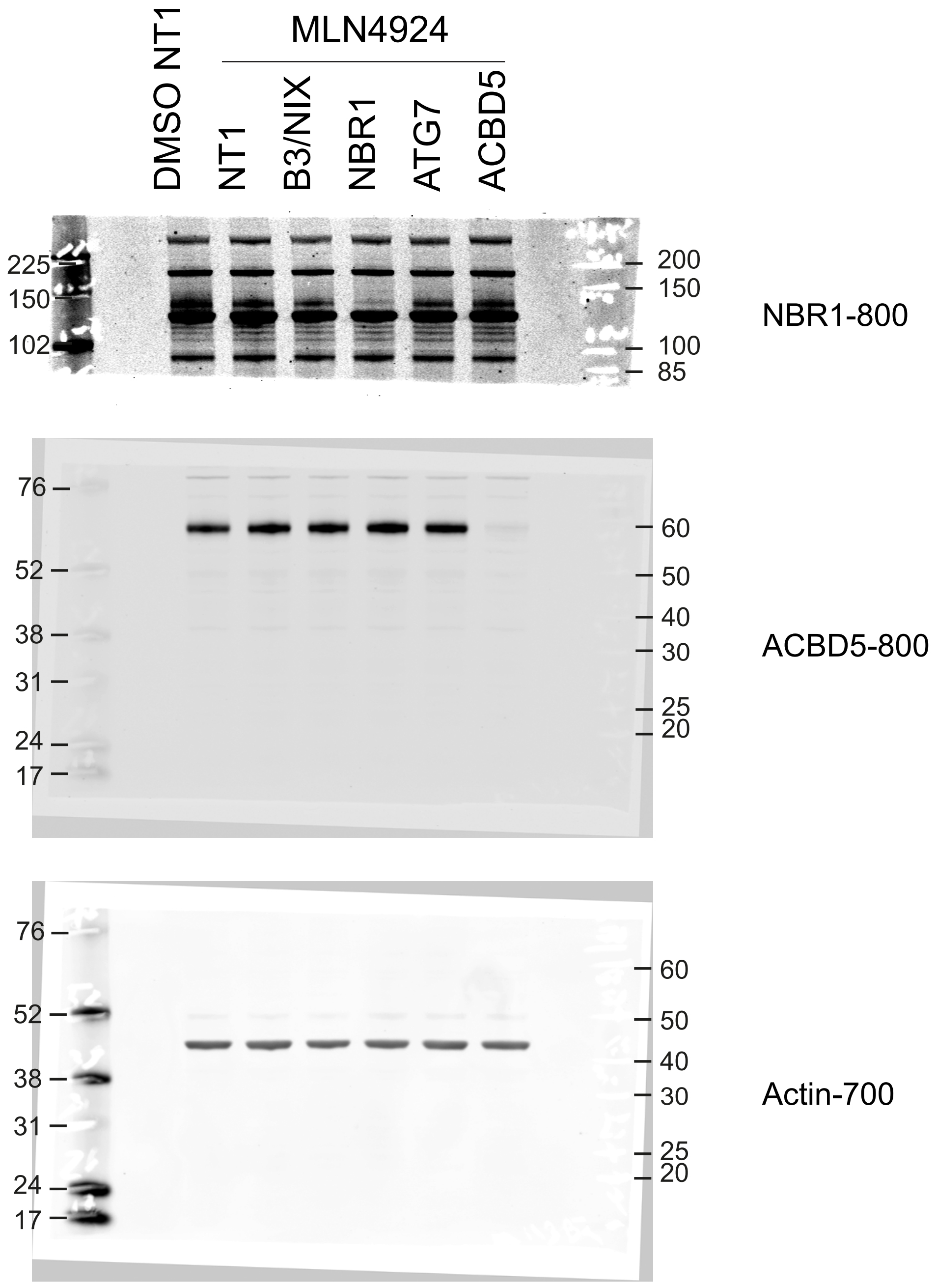

Source Data: Figure 2 H: Blot 1

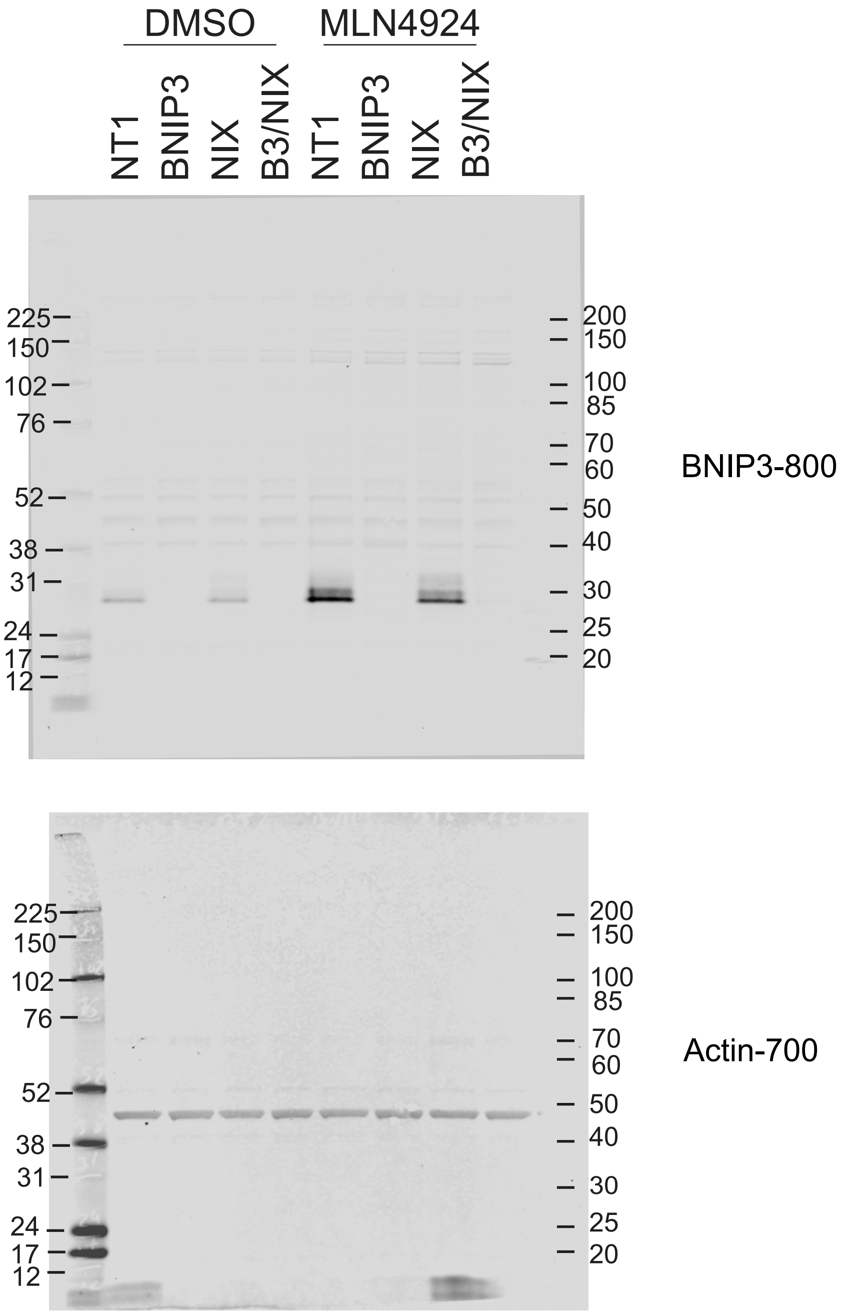

Source Data: Figure 2 H: Blot 2

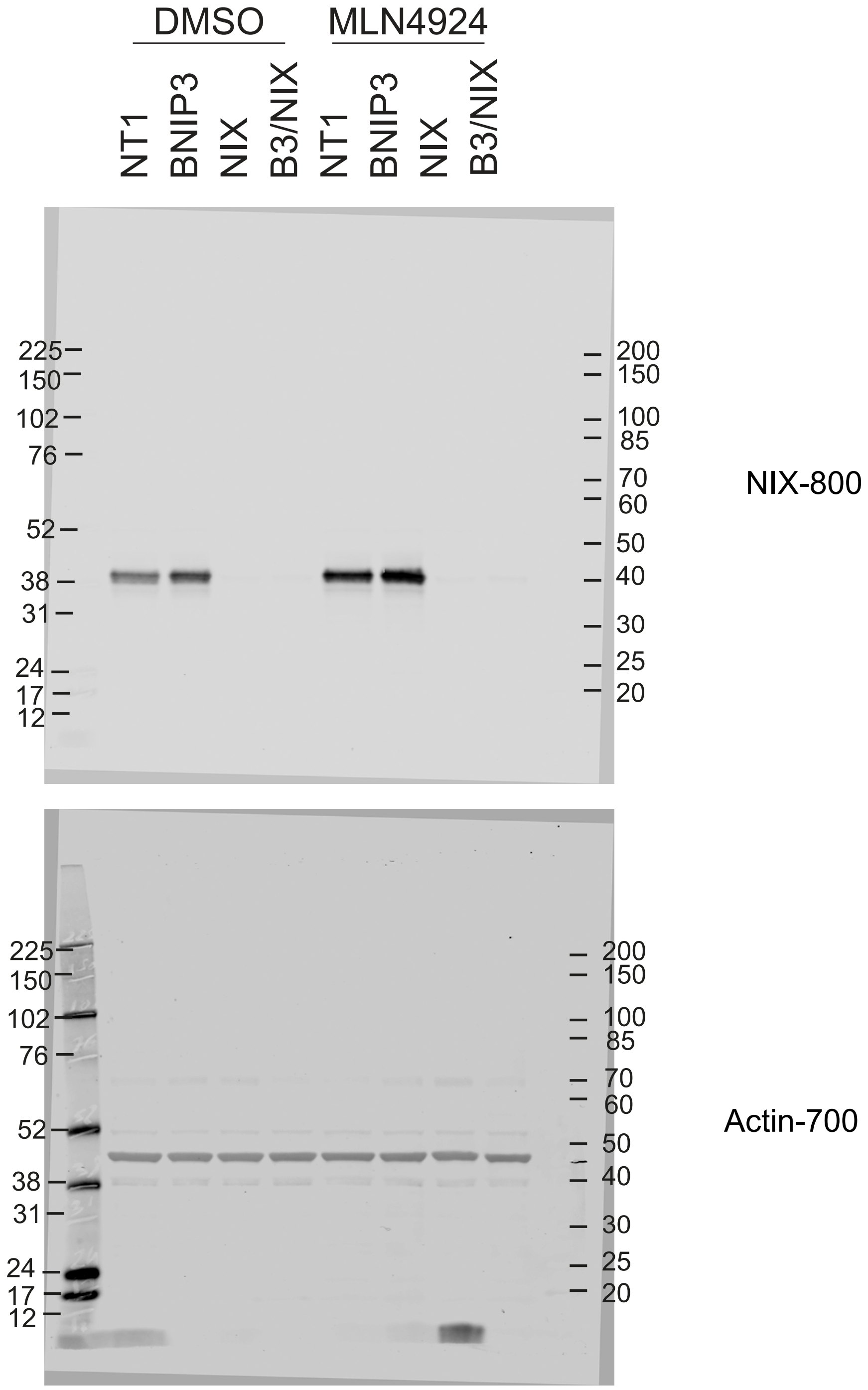

Supplement: Supplementary file 2 [file LSA-2022-01825_SdataF2.pdf]
